# Efficacy and Safety of Lisocabtagene Maraleucel in Relapsed or Refractory Large B-Cell Lymphoma: A Product-Specific Systematic Review and Meta-Analysis of Clinical Trials and Real-World Studies †

**DOI:** 10.3390/hematolrep18040043

**Published:** 2026-06-23

**Authors:** Jerry Qi, Daniel Park, Nidhi Kejriwal, Austin Yang, Kareem Latif, Sarkis Dagley, Mojtaba Akhtari

**Affiliations:** 1 Department of Medicine, Loma Linda University Medical Center, 11234 Anderson St., Loma Linda, CA 92354, USA; 2 Department of Medicine, University of California, San Francisco, Fresno Campus, 155 N. Fresno Street, Fresno, CA 93701, USA; 3 School of Medicine, University of California, Riverside, 900 University Ave., Riverside, CA 92521, USA; 4 College of Medicine, University of Central Florida, 6850 Lake Nona Blvd., Orlando, FL 32827, USA; 5 Division of Transplant, Cellular Therapy, and Hematological Malignancies, Department of Medicine, Loma Linda University Medical Center, 11234 Anderson St., Loma Linda, CA 92354, USA; makhtari@llu.edu

**Keywords:** lisocabtagene maraleucel, liso-cel, large B-cell lymphoma, CAR-T therapy, systematic review, meta-analysis

## Abstract

Background/Objectives: Lisocabtagene maraleucel (liso-cel) is a CD19-directed chimeric antigen receptor T-cell (CAR-T) therapy approved for relapsed or refractory large B-cell lymphoma (R/R LBCL). However, most published meta-analyses of CAR-T therapy in LBCL pool data across products, limiting product-specific interpretation. Methods: We conducted a systematic review and meta-analysis of clinical trials and retrospective real-world studies evaluating liso-cel monotherapy in R/R LBCL. The primary endpoint was the overall response rate (ORR). Secondary endpoints included complete response (CR), incidence of grade ≥ 3 adverse events, including cytokine release syndrome (CRS) and immune effector cell-associated neurotoxicity syndrome (ICANS), overall mortality rate (OMR), disease progression-related mortality, and adverse event-related mortality. Pooled proportions were estimated using random-effects models. Results: Eleven studies including 1206 patients were analyzed, comprising five clinical trials and six real-world retrospective cohorts. The pooled ORR was 78%, and the pooled CR rate was 60%. The pooled OMR was 38%, with a disease progression-related mortality of 28% and an adverse event-related mortality of 4%. Severe (grade ≥ 3) CRS and ICANS occurred in 2% and 8%, respectively. Severe (grade ≥ 3) hematologic toxicities were frequent, particularly neutropenia, thrombocytopenia, and anemia. Conclusions: Liso-cel monotherapy demonstrated high pooled response rates and low pooled incidences of severe CRS and ICANS across clinical trials and real-world settings in R/R LBCL. Severe ICANS, although uncommon, remains clinically meaningful, and severe hematologic toxicities were frequent and warrant careful monitoring and supportive care. These findings provide product-specific benchmarks for liso-cel in R/R LBCL.

## 1. Introduction

Large B-cell lymphoma (LBCL) is the most common subtype of non-Hodgkin lymphoma, accounting for roughly one-third of worldwide cases annually. It is most common in older adults, with the median age at diagnosis in the mid-60s [1]. Frontline therapy for LBCL remains centered on R-CHOP (rituximab, cyclophosphamide, doxorubicin, vincristine, and prednisone), with approximately 60% of patients achieving long-term remission. Despite the success of standard-of-care immunochemotherapy, approximately 10–15% of patients have primary refractory disease, and a further 20–30% of patients experience relapse with poor outcomes [1,2].

Therefore, relapsed or refractory (R/R) LBCL remains a major therapeutic challenge. The curative approach for R/R disease traditionally involves high-dose platinum-based chemotherapy followed by autologous stem cell transplantation (ASCT) for eligible patients [1,3]. Around 50% of patients respond to salvage chemotherapy and eventually undergo ASCT, with a cure rate of 25 to 35% [1]. However, over half of the patients with R/R LBCL are either ineligible for ASCT due to advanced age, medical comorbidities, or chemo-resistant disease or have a relapse after ASCT, leaving a significant population with limited therapeutic options.

The emergence of chimeric antigen receptor (CAR) T-cell therapies has transformed the landscape for R/R LBCL and offers the potential for deep and durable remissions even in highly pretreated and high-risk populations. There are currently three FDA-approved CAR-T therapies for R/R LBCL, including axicabtagene ciloleucel (axi-cel), tisagenlecleucel (tisa-cel), and lisocabtagene maraleucel (liso-cel). While each product shares the same CD19 target, they differ in costimulatory domains, T-cell composition, and associated toxicity profiles [4].

Liso-cel is a novel autologous CD19-directed CAR-T product for R/R LBCL. In the pivotal TRANSCEND NHL 001 trial, liso-cel produced high objective and complete response rates (CRs) with low rates of severe (grade ≥ 3) cytokine release syndrome (CRS) and immune effector cell-associated neurotoxicity syndrome (ICANS) as a third-line and later therapy [5]. Subsequent investigations, including the phase 3 TRANSFORM trial in second-line therapy, have also shown similar efficacy and safety [6].

As liso-cel has increasingly moved into general clinical practice, its use has expanded to include older patients and those with significant comorbidities, populations that are usually underrepresented in prospective studies. However, most existing systematic reviews and meta-analyses evaluate CAR-T therapies collectively across different products. Because CAR-T constructs differ in costimulatory domains, T-cell composition, manufacturing processes, and toxicity profiles, pooling outcomes across products limits the ability to generate product-specific conclusions regarding efficacy and toxicity.

To address this gap in the existing literature, we conducted a systematic review and meta-analysis to characterize the efficacy and safety of liso-cel in patients with R/R LBCL. By integrating evidence from both prospective clinical trials and retrospective real-world studies, this analysis aims to provide a comprehensive and product-specific assessment of treatment outcomes across diverse clinical settings.

## 2. Methods

### 2.1. Search Strategy

**Figure 1:**
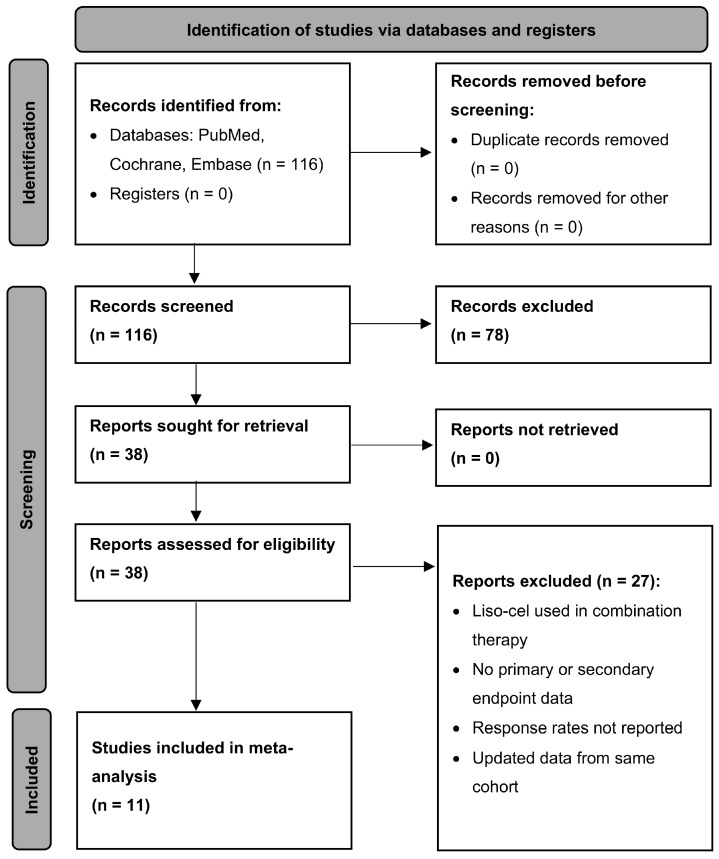
PRISMA flow diagram of study selection.

We conducted a literature search using the PICOS (Population, Interventions, Comparators, Outcomes, and Study Design) framework. This systematic review and meta-analysis was conducted in accordance with the Preferred Reporting Items for Systematic Reviews and Meta-Analyses (PRISMA) 2020 guidelines [7]. The review was not prospectively registered. PubMed, Embase, and Cochrane databases were used to identify studies reporting liso-cel monotherapy in LBCL. The literature search was last updated on 10 May 2025. Eligible study designs included single- and multicenter clinical trials and retrospective studies. Search terms included: “lisocabtagene maraleucel”, “liso-cel”, “monotherapy”, “large B-cell lymphoma”, “LBCL”, and “relapsed refractory”. Inclusion criteria included clinical trials and studies describing liso-cel monotherapy in LBCL. Exclusion criteria included studies and trials utilizing liso-cel in other disease settings and liso-cel used in combination therapies. Case reports, literature reviews, and letters to the editor were also excluded. Study selection was performed independently by two authors, and any discrepancies were resolved by a third reviewer. Data extraction was completed independently by two authors and reviewed by a third author. Figure 1 displays the study selection process.

### 2.2. Risk-of-Bias Assessment

Risk of bias for retrospective observational studies was assessed using a modified Newcastle–Ottawa Scale adapted for single-arm observational CAR-T studies [8]. The scale evaluated selection, comparability, and outcome assessment domains, with a maximum score of 9. Because the included retrospective studies were largely single-arm cohorts, no star was awarded for the non-exposed cohort item. Comparability was scored only when age and/or other clinically relevant prognostic factors were adjusted for or analyzed. Studies were categorized as low risk of bias for scores of 7–9, moderate risk for scores of 4–6, and high risk for scores of 0–3.

### 2.3. Primary and Secondary Outcomes

The primary endpoint of the study was the overall response rate (ORR). Secondary endpoints were complete response (CR), overall mortality rate (OMR; all-cause mortality), disease progression-related mortality, adverse event-related mortality, reported incidence of grade ≥ 3 CRS and ICANS, and reported incidence of other grade ≥ 3 adverse events. OMR was defined as all-cause mortality during the reported study follow-up period. Cause-specific mortality was abstracted as reported by individual studies and categorized as mortality due to disease progression, adverse events, or unknown causes. Adverse event grading criteria were abstracted as reported in each study. CRS and ICANS grading criteria were not uniformly specified across all studies, whereas hematologic toxicities were most commonly reported as grade ≥ 3 events. Grading criteria, reporting windows, and definitions of prolonged cytopenia were not uniformly specified across all studies.

### 2.4. Statistical Methods

Meta-analyses of proportions were performed to estimate the pooled proportions of ORR, CR, OMR, and adverse event outcomes across included studies. Because heterogeneity between studies was expected, all analyses were performed using a random-effects model. No variance-stabilizing transformation, including Freeman–Tukey double arcsine transformation, was applied when pooling proportions. For low-frequency outcomes, pooled estimates were interpreted cautiously because sparse events and studies with zero or few events may influence variance estimation and confidence interval width. Heterogeneity was evaluated using the I^2^ statistic, which represents the percentage of variability attributable to heterogeneity rather than to sampling error. Outcomes with I^2^ ≥ 60% were considered to represent high heterogeneity. Publication bias was assessed qualitatively based on study design, sample size, publication type, and completeness of outcome reporting. Individual 95% confidence intervals were calculated using Clopper–Pearson intervals for binomial proportions. Meta-analyses were performed using the *meta* package in *R*.

## 3. Results

Eleven studies, including five clinical trials and six real-world retrospective studies, were selected for this meta-analysis, including 1206 patients. Table 1 describes the baseline characteristics of the included patients. In most studies, liso-cel was administered according to the protocol established in the TRANSCEND NHL 001 trial [5]. The efficacy and safety endpoints analyzed across studies were ORR, CR, OMR, and grade ≥ 3 AEs.

Risk of bias was assessed for the six retrospective observational studies using a modified Newcastle–Ottawa Scale (Supplementary Table S1). Three studies were judged to have a low risk of bias, with scores ranging from 7 to 8 out of 9, and three were judged to have a moderate risk of bias, each scoring 6 out of 9. The selection and outcome assessment domains were generally adequate, as most studies used clearly defined institutional or registry-based cohorts and reported objective post-infusion efficacy and safety outcomes. The main limitations were the lack of non-exposed comparator cohorts, limited adjusted analyses for age or other prognostic factors, and restricted methodological detail in abstract-only reports. No retrospective study was judged to have a high risk of bias.

### 3.1. Evaluation of Efficacy

**Figure 2:**
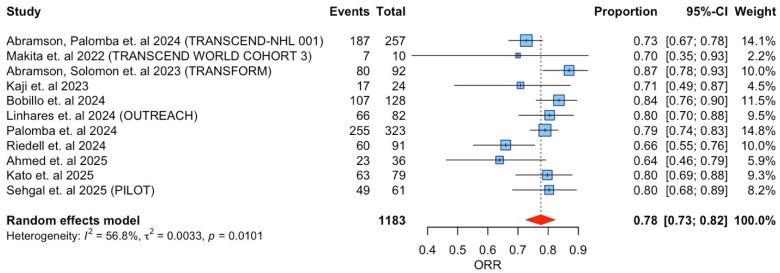
Forest plot of pooled ORR: 78% (95% CI 73–82%, I^2^ = 56.8%, *p* = 0.0101). Data were derived from contributing studies [5,6,9,10,11,12,13,14,15,16,17]. Blue squares represent individual study estimates, with square size proportional to study weight. Horizontal lines represent 95% confidence intervals. The red diamond represents the pooled random-effects estimates. The dashed vertical line represents the pooled effect estimate.

**Figure 3:**
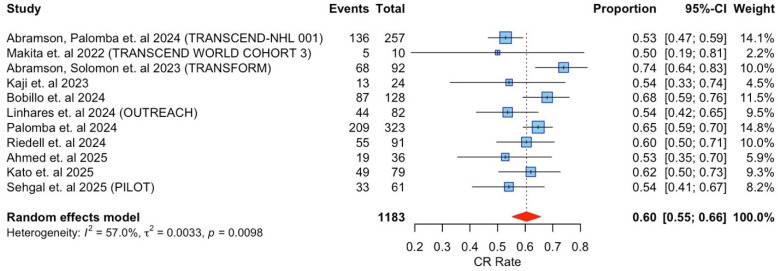
Forest plot of pooled CR: 60% (95% CI 55–66%, I^2^ = 57.0%, *p* = 0.0098). Data were derived from contributing studies [5,6,9,10,11,12,13,14,15,16,17]. Blue squares represent individual study estimates, with square size proportional to study weight. Horizontal lines represent 95% confidence intervals. The red diamond represents the pooled random-effects estimates. The dashed vertical line represents the pooled effect estimate.

The ORR was assessed in all 11 studies. The pooled ORR was 78% (95% confidence interval [CI] 73–82%, I^2^ = 56.8%, *p* = 0.0101) (Figure 2). CR was also assessed in all 11 studies. The pooled CR rate was 60% (95% CI 55–66%, I^2^ = 57.0%, *p* = 0.0098) (Figure 3).

### 3.2. Evaluation of Mortality

**Figure 4:**
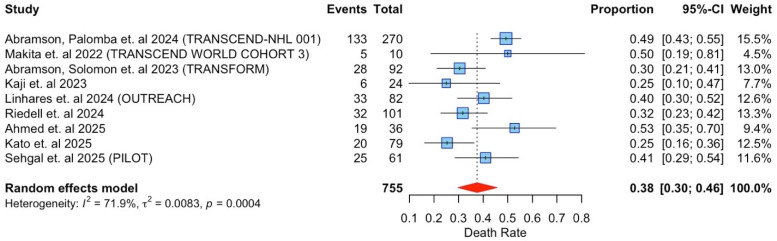
Forest plot of pooled overall mortality rate: 38% (95% CI 30–46%, I^2^ = 71.9%, *p* = 0.0004). Data were derived from contributing studies [5,6,9,10,12,14,15,16,17]. Blue squares represent individual study estimates, with square size proportional to study weight. Horizontal lines represent 95% confidence intervals. The red diamond represents the pooled random-effects estimates. The dashed vertical line represents the pooled effect estimate.

**Figure 5:**
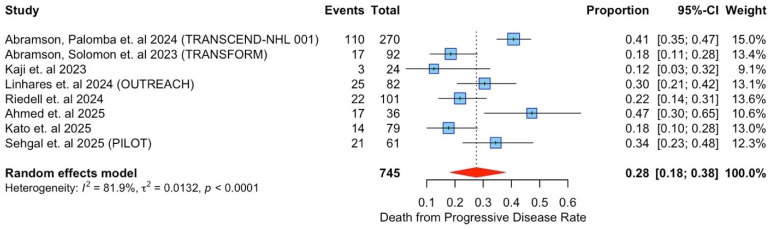
Forest plot of pooled disease progression-related mortality rate: 28% (95% CI 18–38%, I^2^ = 81.9%, *p* < 0.0001). Data were derived from contributing studies [5,6,10,12,14,15,16,17]. Blue squares represent individual study estimates, with square size proportional to study weight. Horizontal lines represent 95% confidence intervals. The red diamond represents the pooled random-effects estimates. The dashed vertical line represents the pooled effect estimate.

**Figure 6:**
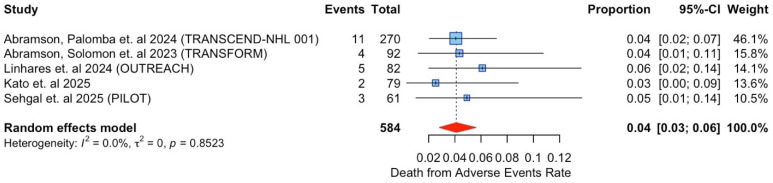
Forest plot of pooled adverse event-related mortality rate: 4% (95% CI 3–6%, I^2^ = 0.0%, *p* = 0.8523). Data were derived from contributing studies [5,6,12,16,17]. Blue squares represent individual study estimates, with square size proportional to study weight. Horizontal lines represent 95% confidence intervals. The red diamond represents the pooled random-effects estimates. The dashed vertical line represents the pooled effect estimate.

The overall mortality rate (OMR) was analyzed in nine studies. The pooled OMR was 38% (95% CI 30–46%, I^2^ = 71.9%, *p* = 0.0004) (Figure 4). The disease progression-related mortality rate was reported in eight studies, with a pooled rate of 28% (95% CI 18–38%, I^2^ = 81.9%, *p* < 0.0001) (Figure 5). Adverse event-related mortality was assessed in five studies, with a pooled rate of 4% (95% CI 3–6%, I^2^ = 0.0%, *p* = 0.8523) (Figure 6). Mortality from unknown causes was analyzed in four studies, with a pooled rate of 2% (95% CI 0–4%, I^2^ = 0.0%, *p* = 0.5048).

### 3.3. Evaluation of Adverse Events

**Figure 7:**
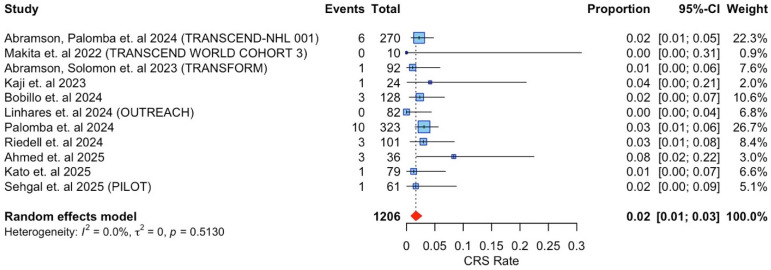
Forest plot of pooled grade ≥ 3 CRS: 2% (95% CI 1–3%, I^2^ = 0.0%, *p* = 0.5130). Data were derived from contributing studies [5,6,9,10,11,12,13,14,15,16,17]. Blue squares represent individual study estimates, with square size proportional to study weight. Horizontal lines represent 95% confidence intervals. The red diamond represents the pooled random-effects estimates. The dashed vertical line represents the pooled effect estimate.

**Figure 8:**
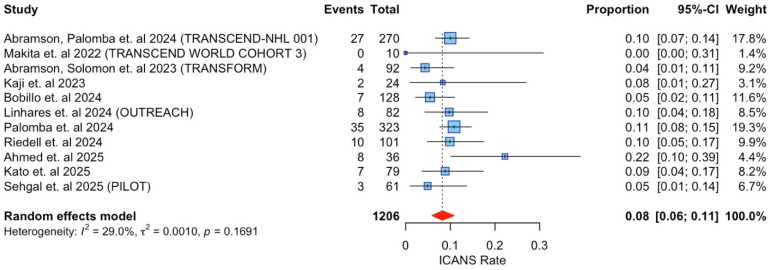
Forest plot of pooled grade ≥ 3 ICANS: 8% (95% CI 6–11%, I^2^ = 29.0%, *p* = 0.1691). Data were derived from contributing studies [5,6,9,10,11,12,13,14,15,16,17]. Blue squares represent individual study estimates, with square size proportional to study weight. Horizontal lines represent 95% confidence intervals. The red diamond represents the pooled random-effects estimates. The dashed vertical line represents the pooled effect estimate.

Grade ≥ 3 CRS and ICANS were analyzed. The pooled incidence of grade ≥ 3 CRS was 2% (95% CI 1–3%, I^2^ = 0.0%, *p* = 0.5130), and the pooled incidence of grade ≥ 3 ICANS was 8% (95% CI 6–11%, I^2^ = 29.0%, *p* = 0.1691) (Figure 7 and Figure 8).

Other common grade ≥ 3 AEs were also evaluated. The pooled incidence rates were as follows: neutropenia 72% (95% CI 49–90%, I^2^ = 88.2%, *p* < 0.0001), thrombocytopenia 40% (95% CI 18–63%, I^2^ = 88.3%, *p* < 0.0001), anemia 39% (95% CI 17–62%, I^2^ = 88.6%, *p* < 0.0001), leukopenia 28% (95% CI 5–59%, I^2^ = 89.7%, *p* < 0.0001), lymphopenia 15% (95% CI 0–55%, I^2^ = 88.4%, *p* = 0.0002), febrile neutropenia 10% (95% CI 0–33%, I^2^ = 0.0%, *p* = 0.3711).

## 4. Discussion

In this systematic review and meta-analysis of 11 studies including 1206 patients, liso-cel monotherapy demonstrated high pooled response rates across both clinical trials and real-world settings for R/R LBCL. Although prior studies have demonstrated the efficacy of liso-cel, reported response outcomes vary across individual studies. This meta-analysis therefore evaluates the consistency of these outcomes across diverse patient populations. The pooled ORR of 78% and CR rate of 60% compare favorably with the historically poor outcomes seen in the pre-CAR-T era [18,19].

Our pooled efficacy estimates are consistent with those reported in liso-cel’s major prospective studies. In TRANSCEND NHL 001, which evaluated third-line and later therapy, liso-cel achieved an ORR of 73% and a CR rate of 53%. Longer-term follow-up also showed a median duration of response (DOR) of 23.1 months and estimated 2-year overall survival (OS) of 50.5% [5]. In the second-line setting, TRANSFORM demonstrated improved event-free survival (EFS), progression-free survival (PFS), and a higher ORR (87%) and CR rate (74%) compared with standard salvage chemotherapy followed by ASCT [6]. In PILOT, transplant-ineligible patients, who are often older and medically frail, also achieved high response rates (ORR 80%, CR 54%), with a median DOR of 23.3 months and median OS not reached at final analysis [16]. The retrospective second-line CIBMTR registry study by Bobillo et al. also reported ORR and CR rates that were broadly consistent with the overall pooled estimates. These earlier-line data suggest that response outcomes were generally aligned with the overall pooled rates. Similarly, studies reflecting third-line or later treatment, including OUTREACH and Palomba et al., reported ORR and CR rates that were generally similar to the overall pooled estimates. The remaining real-world cohorts included mixed treatment-line populations, limiting consistent categorization across all studies and precluding formal stratified meta-analysis by treatment line. Although survival and durability endpoints such as DOR, OS, EFS, and PFS are important for interpreting long-term CAR-T benefit, they were not consistently reported across real-world cohorts and were therefore not pooled. Nevertheless, the pooled ORR of 78% and the CR rate of 60% in our analysis are consistent with these prospective and real-world data, supporting the consistent efficacy of liso-cel across both clinical trial and real-world populations.

Real-world cohorts represented a substantial portion of this meta-analysis and strengthened the clinical relevance of our findings. Several included retrospective studies enrolled patients who would not have met eligibility criteria for pivotal trials, including those with advanced age, significant comorbidities, or other high-risk features. Across the included studies, the median age ranged from 57 to 74 years. Several real-world cohorts included older populations, including Bobillo et al. with a median age of 73 years, Palomba et al. at 70 years, and Riedell et al. at 71 years. Notably, older patients were also represented in the prospective PILOT study, which enrolled transplant-ineligible patients and reported a median age of 74 years. Despite the inclusion of these broader patient populations, the pooled ORR and CR remained high and generally consistent with outcomes reported in clinical trials. However, differences between protocol-defined clinical trial populations and broader real-world cohorts should be considered when interpreting pooled estimates. Taken together, these findings suggest that liso-cel retains clinically meaningful activity in general clinical practice and that its benefit may extend beyond highly selected clinical trial populations.

Another key finding from our analysis is the low pooled incidences of severe (grade ≥ 3) CRS and ICANS. The pooled incidence of grade ≥ 3 CRS was 2%, while grade ≥ 3 ICANS occurred in 8% of patients, similar to rates reported in major clinical trials. Severe ICANS, although relatively infrequent, is clinically important because it can substantially affect post-infusion monitoring, need for higher-acuity care, and neurologic recovery. Its management usually consists of supportive care and corticosteroids, although the optimal timing, dose, and duration of corticosteroid therapy remain unstandardized [20]. At the same time, interpretation of pooled grade ≥ 3 ICANS rates is limited by differences in grading systems across studies. Pooled incidence alone cannot fully capture the variability in symptom severity, duration, reversibility, need for ICU-level care, or longer-term neurologic sequelae [20]. Because many included real-world studies did not provide detailed ICANS data, specific ICANS presentation and management information could not be pooled or compared. However, the low pooled rate of adverse event-related mortality suggests that severe CRS and ICANS are often manageable with monitoring and contemporary supportive care.

The low pooled incidences of severe CRS and ICANS may be interpreted in the context of product-level biological and manufacturing differences among approved CD19-directed CAR-T therapies. Approved CD19-directed CAR-T products share the same FMC63-derived antigen-binding domain but differ in costimulatory domain, gene transfer method, and cellular composition [21,22]. While axi-cel incorporates a CD28 costimulatory domain, which drives rapid, high-magnitude T-cell expansion, liso-cel and tisa-cel use a 4-1BB (CD137) costimulatory domain, which promotes more gradual expansion kinetics and greater long-term persistence. These differences in T-cell expansion kinetics, phenotype, and persistence have been associated with variation in inflammatory toxicity patterns [23]. Consistent with this biologic framework, a prior systematic review reported lower rates of grade ≥ 3 adverse events with 4-1BB-containing CAR-T products compared with CD28-containing products [24]. In addition, liso-cel uses a defined composition process in which CD4^+^ and CD8^+^ T cells are separately selected, transduced, expanded, and recombined at a fixed 1:1 ratio. This approach may reduce between-patient variability in the infused product and yield less-differentiated T-cell populations [25,26]. In comparison, axi-cel and tisa-cel are manufactured from unselected T-cell populations with variable CD4:CD8 ratios [27]. These structural and manufacturing distinctions may help contextualize the low pooled incidences of severe CRS and ICANS observed with liso-cel, although cross-product comparisons remain limited by differences in patient populations, trial designs, toxicity grading, and supportive care practices.

In contrast, liso-cel was associated with frequent severe (grade ≥ 3) hematologic toxicities. Grade ≥ 3 neutropenia (72%), thrombocytopenia (40%), anemia (39%), and leukopenia (28%) were common across studies. These cytopenias, now commonly described as immune effector cell-associated hematotoxicity (ICAHT), are increasingly recognized as multifactorial toxicities that extend beyond the direct myelosuppressive effects of lymphodepleting chemotherapy. Early cytopenias within 30 days are primarily attributed to lymphodepletion and CRS-related inflammatory stress, whereas prolonged cytopenias beyond 30 days reflect a more complex pathophysiology involving impaired bone marrow reserve, sustained inflammatory signaling, and immune dysregulation [28,29]. Proinflammatory cytokines released during CAR-T cell expansion, particularly IFN-γ and IL-6, can drive hematopoietic stem cell apoptosis, disrupt the bone marrow stromal niche, and suppress hematopoietic recovery [28,30]. Cumulative cytotoxic effects from prior lines of therapy, tumor burden, and bridging therapy exposure further contribute to the severity and duration of cytopenias in heavily pretreated patients. The CAR-HEMATOTOX scoring model, which incorporates markers of hematopoietic reserve and baseline inflammation, has been shown to predict prolonged severe neutropenia after CAR-T therapy and may facilitate risk-adapted management [31]. Future studies of supportive care strategies, including optimized growth factor use, thrombopoietin receptor agonists, and autologous stem cell boosts, may help mitigate complications related to these hematologic toxicities [32]. Despite the frequency of severe hematologic adverse events, pooled adverse event-related mortality remained low at 4%.

Substantial heterogeneity was observed for hematologic toxicities, with I^2^ > 80% across most events. This variability likely reflects both methodological and clinical differences across studies. Methodological and reporting differences, including prospective versus retrospective study design, treatment line, follow-up duration, adverse event reporting windows, and cytopenia definitions, may have contributed to the observed variability. Clinical differences, including baseline marrow reserve, prior therapy burden, bridging therapy exposure, lymphodepletion approach, and disease burden, may also have contributed. In addition, hematologic toxicity estimates were derived from a limited number of contributing studies. Therefore, high I^2^ values for cytopenia outcomes may reflect both true clinical heterogeneity and statistical instability from small numbers of studies with variable reporting practices. For example, the Kaji et al. study was an abstract-only real-world cohort that contributed to hematologic toxicity estimates but provided limited detail on adverse event reporting windows and grading criteria, which may have led to overestimation or underestimation of pooled cytopenia rates. Larger studies with standardized adverse event reporting and sufficient study-level or patient-level data are needed to better evaluate sources of heterogeneity in hematologic toxicities after liso-cel.

Despite high response rates, pooled overall mortality remained substantial at 38%, with most deaths attributable to disease progression (28%). This likely reflects a combination of primary nonresponse and early relapse. Substantial heterogeneity was also observed in mortality outcomes, particularly overall mortality and mortality due to disease progression. This variability likely reflects differences in follow-up duration, treatment line, baseline disease risk, disease status before lymphodepletion, bridging therapy response, and inclusion of trial-ineligible patients in real-world cohorts. In addition, mortality endpoints may not have been uniformly defined or attributed across studies, with variation in whether deaths were reported as all-cause mortality, disease progression-related mortality, or adverse event-related mortality. Further studies with longer follow-up, standardized mortality definitions, standardized cause-of-death attribution, and more-complete reporting of baseline risk factors are needed to better evaluate sources of heterogeneity in mortality outcomes.

The therapeutic landscape for R/R LBCL has evolved considerably since the initial approval of CAR-T products in the third-line or later setting, as CAR-T therapy has moved into earlier lines of treatment. In the second-line setting, ZUMA-7 with axi-cel and TRANSFORM with liso-cel demonstrated improved EFS compared with salvage chemotherapy followed by ASCT, whereas BELINDA did not demonstrate superiority of tisa-cel over standard care [6,33,34]. These results support the use of both axi-cel and liso-cel in the second-line setting before salvage chemotherapy and ASCT, shifting the treatment paradigm for patients with primary refractory or early relapsed LBCL. Despite these advances, disease progression or relapse after CAR-T therapy remains common, and outcomes after post-CAR-T relapse are poor [35]. Emerging retrospective data have shown responses from polatuzumab- or lenalidomide-based therapies in post-CAR-T patients, while CD20 × CD3-bispecific antibodies, including epcoritamab and glofitamab, have expanded available treatment options and introduced new sequencing considerations [35,36]. Because CAR-T products target CD19 and CD20 × CD3-bispecific antibodies target CD20, these platforms engage non-overlapping antigens, which may allow for complementary or sequential use. However, optimal sequencing remains undefined, highlighting the need for product-specific benchmarks such as those provided in the present analysis.

The pooled efficacy and safety estimates from the present analysis provide product-specific benchmarks for liso-cel that may help inform treatment selection for R/R LBCL. These findings should be interpreted alongside the limitations of cross-study heterogeneity, the absence of direct comparative analyses with other CAR-T products, and the risk-of-bias profile of the retrospective studies. Among the six retrospective studies, three were judged to have a low risk of bias, and three were judged to have a moderate risk of bias, with no retrospective study judged to have a high risk of bias. However, because most retrospective cohorts were single-arm studies without comparator groups and adjusted analyses were limited, residual confounding remains possible and causal interpretation of pooled efficacy and toxicity estimates is limited. Nevertheless, the combination of high response rates and low pooled incidences of severe CRS and ICANS across clinical trials and real-world studies supports liso-cel as an effective treatment option for R/R LBCL.

## 5. Limitations

Due to the limited literature available on liso-cel treatment for R/R LBCL, both retrospective and prospective studies were included, which introduced considerable heterogeneity in study design, patient populations, follow-up duration, and outcome reporting. This analysis also pooled studies across different treatment lines, including second-line and third-line or later settings, which may have contributed to variability in outcomes. Follow-up duration also varied across included studies, with median follow-up ranging from 6.2 to 19.9 months, which may have influenced overall mortality and cause-specific mortality estimates. Although subgroup analyses and meta-regression could further explore heterogeneity, these analyses were not performed because the number of available studies for several outcomes was limited, and key covariates were inconsistently reported across studies. Such analyses may have been underpowered in the present dataset and should be explored in future studies with larger datasets. This systematic review was not prospectively registered in PROSPERO, which may reduce methodological transparency and should be considered when interpreting the findings.

Publication and reporting bias may have influenced pooled estimates, particularly because several real-world studies were available only as conference abstracts. These conference abstract data were included to capture emerging real-world evidence but were interpreted cautiously because of limited methodological detail, outcome adjudication, follow-up completeness, and adjustment for confounding. Formal publication bias assessment using funnel plots or Egger’s test was not performed because many outcomes included a small number of studies and several endpoints were pooled proportions with sparse event counts, limiting the reliability and interpretability of these methods. Because adverse event grading criteria, reporting windows, and definitions of prolonged cytopenia varied across studies, pooled grade ≥ 3 cytopenia rates should be interpreted as approximate product-specific estimates rather than precise institutional benchmarks. Although risk of bias was assessed using a modified Newcastle–Ottawa Scale, several retrospective studies were single-arm analyses without comparator cohorts, and adjusted analyses for age or other prognostic factors were inconsistently reported. These limitations reduce causal interpretability and may contribute to between-study heterogeneity. Furthermore, liso-cel was not directly compared with other CAR-T products, limiting cross-product comparative conclusions. Nonetheless, this product-specific analysis provides clinically valuable pooled estimates for liso-cel monotherapy in R/R LBCL.

## 6. Conclusions

This systematic review and meta-analysis provides a product-specific pooled analysis of liso-cel monotherapy in patients with R/R LBCL across both clinical trials and real-world settings, offering clinically relevant benchmarks for liso-cel efficacy and safety. Liso-cel demonstrated high pooled response rates, with an ORR of 78% and a CR rate of 60%, along with low pooled incidences of severe CRS and ICANS. Severe ICANS, although uncommon, remains clinically meaningful, and severe hematologic toxicities were frequent and warrant careful monitoring and supportive care. These findings support liso-cel as an important treatment option for R/R LBCL. Future studies with longer follow-up, standardized toxicity and mortality reporting, and more-complete survival endpoint reporting are needed to better define durability of response, optimal sequencing, and comparative outcomes in the evolving LBCL treatment landscape.

## Figures and Tables

**Table 1:** Baseline demographic characteristics of R/R LBCL patients treated with liso-cel monotherapy.

Study (Year)	*N*	Median Age (Years)	Prior Lines of Therapy, Median	ECOG 0–1, *n* (%)	ECOG ≥ 2, *n* (%)	HGBCL, *n* (%)	Previous HSCT, *n* (%)	Median Follow-Up, Months
Abramson, Palomba et al., 2024 [5]	270	63	3	266 (99%)	4 (1%)	–	94 (35%)	19.9
Makita et al., 2022 [9]	10	57	3	10 (100%)	–	–	2 (20%)	12.5
Abramson, Solomon et al., 2023 [6]	92	60	1	92 (100%)	–	–	–	17.5
Kaji et al., 2023 [10]	24	64.5	4	21 (88%)	3 (13%)	2 (8%)	8 (33%)	7.7
Bobillo et al., 2024 [11]	128	73	2	102 (80%)	5 (4%)	18 (14%)	–	6.2
Linhares et al., 2024 [12]	82	66	2	82 (100%)	–	–	2 (2%)	10.6
Palomba et al., 2024 [13]	323	70	3	274 (85%)	18 (6%)	38 (12%)	–	7.4
Riedell et al., 2024 [14]	101	71	3	72 (71%)	–	7 (7%)	–	15.5
Ahmed et al., 2025 [15]	36	62	3	27 (75%)	4 (11%)	9 (25%)	–	12.0
Sehgal et al., 2025 [16]	61	74	1	45 (74%)	16 (26%)	18 (30%)	–	18.2
Kato et al., 2025 [17]	79	63	3	–	–	4 (5%)	–	7.0

## Data Availability

The data presented in this study are derived from published articles cited in the manuscript.

## Supplementary Materials

The following supporting information can be downloaded at https://www.mdpi.com/article/10.3390/hematolrep18040043/s1. Supplementary Table S1: Modified Newcastle–Ottawa Scale risk-of-bias assessment for included retrospective observational studies.

## References

[1] Sehn L.H., Salles G. (2021). Diffuse large B-cell lymphoma. N. Engl. J. Med..

[2] Barraclough A., Hawkes E., Sehn L.H., Smith S.M. (2024). Diffuse large B-cell lymphoma. Hematol. Oncol..

[3] Chaganti S., Fox C.P., Maybury B.D., Burton C., Barrington S.F., Illidge T., Kalakonda N., Eyre T.A., McKay P., Kuhnl A. (2025). Management of relapsed or refractory large B-cell lymphoma: A British Society for Haematology Guideline. Br. J. Haematol..

[4] Smith R., Shen R. (2023). Complexities in comparing the impact of costimulatory domains on approved CD19 CAR functionality. J. Transl. Med..

[5] Abramson J.S., Palomba M.L., Gordon L.I., Lunning M., Wang M., Arnason J., Purev E., Maloney D.G., Andreadis C., Sehgal A. (2024). Two-year follow-up of lisocabtagene maraleucel in relapsed or refractory large B-cell lymphoma in TRANSCEND NHL 001. Blood.

[6] Abramson J.S., Solomon S.R., Arnason J., Johnston P.B., Glass B., Bachanova V., Ibrahimi S., Mielke S., Mutsaers P., Hernandez-Ilizaliturri F. (2023). Lisocabtagene maraleucel as second-line therapy for large B-cell lymphoma: Primary analysis of the phase 3 TRANSFORM study. Blood.

[7] Page M.J., McKenzie J.E., Bossuyt P.M., Boutron I., Hoffmann T.C., Mulrow C.D., Shamseer L., Tetzlaff J.M., Akl E.A., Brennan S.E. (2021). The PRISMA 2020 statement: An updated guideline for reporting systematic reviews. BMJ.

[8] Wells G.A., Shea B., O’Connell D., Peterson J., Welch V., Losos M., Tugwell P. The Newcastle-Ottawa Scale (NOS) for Assessing the Quality of Nonrandomised Studies in Meta-Analyses; Ottawa Hospital Research Institute: Ottawa, ON, Canada. https://ohri.ca/en/who-we-are/core-facilities-and-platforms/ottawa-methods-centre/newcastle-ottawa-scale.

[9] Makita S., Yamamoto G., Maruyama D., Asano-Mori Y., Kaji D., Ananthakrishnan R., Ogasawara K., Stepan L., Schusterbauer C., Rettby N. (2022). Phase 2 results of lisocabtagene maraleucel in Japanese patients with relapsed/refractory aggressive B-cell non-Hodgkin lymphoma. Cancer Med..

[10] Kaji D., Watanabe O., Yamaguchi K., Kageyama K., Taya Y., Nishida A., Takagi S., Yamamoto H., Ishiwata K., Asano-Mori Y. (2023). Efficacy of lisocabtagene maraleucel for relapsed or refractory aggressive B cell lymphoma in our hospital. Blood.

[11] Bobillo M.S.O., Kambhampati S., Lee D., Hunter B.D., Egini O., Patel K., Reagan P.M., Bernasconi D., Kim S., Parrilla C.S. (2024). Real-world (RW) outcomes of lisocabtagene maraleucel (liso-cel) as second-line (2L) therapy in patients (pts) with relapsed or refractory (R/R) large B-cell lymphoma (LBCL): First results from the center for international blood and marrow transplant research (CIBMTR) registry. Blood.

[12] Linhares Y., Freytes C.O., Cherry M., Bachier C., Maris M., Hoda D., Varela J.C., Bellomo C., Cross S., Essell J. (2024). OUTREACH: Phase 2 study of lisocabtagene maraleucel as outpatient or inpatient treatment at community sites for R/R LBCL. Blood Adv..

[13] Palomba M.L., Crombie J.L., Nastoupil L.J., Andreadis C., Isufi I., Hunter B., Winter A., Hess B.T., Barta S.K., Frigault M.J. (2024). Multicenter, real-world study in patients with R/R large B-cell lymphoma (LBCL) who received lisocabtagene maraleucel (liso-cel) in the United States (US). Transplant. Cell Ther..

[14] Riedell P.A., Grady C.B., Nastoupil L.J., Luna A., Ahmed N., Maziarz R.T., Hu M., Brower J., Hwang W.T., Schuster S.J. (2025). Lisocabtagene maraleucel for relapsed/refractory large B-cell lymphoma: A cell therapy consortium real-world analysis. Blood Adv..

[15] Ahmed S., Kallam A., Frigault M., Hunter B.D., Patel S.S., Bernasconi D., Kim S., Krimmel T., Liu F.F., Roy D. (2025). Outcomes of standard of care (SOC) lisocabtagene maraleucel (liso-cel) in patients (Pts) with relapsed or refractory large B-cell lymphoma (LBCL) and secondary CNS (sCNS) involvement from the center for international blood and marrow transplant research (CIBMTR) registry. Transplant. Cell Ther..

[16] Sehgal A., Hoda D., Riedell P.A., Ghosh N., Hamadani M., Hildebrandt G.C., Godwin J.E., Reagan P.M., Wagner-Johnston N.D., Essell J. (2025). Lisocabtagene maraleucel for R/R LBCL in patients not intended for HSCT: Final results of the phase 2 PILOT study. Blood Adv..

[17] Kato K., Tatetsu H., Fukushima K., Mimura N., Nakashima Y., Minakata D., Yoshida A., Fujii N., Satake A., Fukuhara N. (2025). Multicenter real-world study in patients with relapsed/refractory large B-cell lymphoma who received lisocabtagene maraleucel; JSCT-CART23 study from Japan study group for cell therapy and transplantation. Transplant. Cell Ther..

[18] Gisselbrecht C., Glass B., Mounier N., Singh Gill D., Linch D.C., Trneny M., Bosly A., Ketterer N., Shpilberg O., Hagberg H. (2010). Salvage regimens with autologous transplantation for relapsed large B-cell lymphoma in the rituximab era. J. Clin. Oncol..

[19] Crump M., Neelapu S.S., Farooq U., Van Den Neste E., Kuruvilla J., Westin J., Link B.K., Hay A., Cerhan J.R., Zhu L. (2017). Outcomes in refractory diffuse large B-cell lymphoma: Results from the international SCHOLAR-1 study. Blood.

[20] Sterner R.C., Sterner R.M. (2022). Immune effector cell associated neurotoxicity syndrome in chimeric antigen receptor-T cell therapy. Front. Immunol..

[21] Ho M., Zanwar S., Paludo J. (2024). Chimeric antigen receptor T-cell therapy in hematologic malignancies: Successes, challenges, and opportunities. Eur. J. Haematol..

[22] Khan A.N., Asija S., Pendhari J., Purwar R. (2024). CAR-T cell therapy in hematological malignancies: Where are we now and where are we heading for?. Eur. J. Haematol..

[23] Cappell K.M., Kochenderfer J.N. (2021). A comparison of chimeric antigen receptors containing CD28 versus 4-1BB costimulatory domains. Nat. Rev. Clin. Oncol..

[24] Zhu Y., Liu K., Rosen S.T., Liu W., Zhu H. (2025). Treatment-related adverse events of chimeric antigen receptor-T therapies for cancers in clinical trials: A systematic review and meta-analysis. eClinicalMedicine.

[25] Abramson J.S., Palomba M.L., Gordon L.I., Lunning M.A., Wang M., Arnason J., Mehta A., Purev E., Maloney D.G., Andreadis C. (2020). Lisocabtagene maraleucel for patients with relapsed or refractory large B-cell lymphomas (TRANSCEND NHL 001): A multicentre seamless design study. Lancet.

[26] Teoh J., Brown L.F. (2022). Developing lisocabtagene maraleucel chimeric antigen receptor T-cell manufacturing for improved process, product quality and consistency across CD19+ hematologic indications. Cytotherapy.

[27] Capolla S., Rasool M., Toffoli G., Dal Bo M. (2025). CAR-T Cell Manufacturing for Hematological and Solid Tumors: From the Preclinical to Clinical Point of View. Cancer Med..

[28] Rejeski K., Jain M.D., Shah N.N., Perales M.A., Subklewe M. (2024). Immune effector cell-associated haematotoxicity after CAR T-cell therapy: From mechanism to management. Lancet Haematol..

[29] Rejeski K., Subklewe M., Locke F.L. (2023). Recognizing, defining, and managing CAR-T hematologic toxicities. Hematol. Am. Soc. Hematol. Educ. Program..

[30] Read J.A., Rouce R.H., Mo F., Mamonkin M., King K.Y. (2023). Apoptosis of Hematopoietic Stem Cells Contributes to Bone Marrow Suppression Following Chimeric Antigen Receptor T Cell Therapy. Transplant. Cell Ther..

[31] Rejeski K., Perez A., Sesques P., Hoster E., Berger C., Jentzsch L., Mougiakakos D., Frölich L., Ackermann J., Bücklein V. (2021). CAR-HEMATOTOX: A model for CAR T-cell-related hematologic toxicity in relapsed/refractory large B-cell lymphoma. Blood.

[32] Jain T., Olson T.S., Locke F.L. (2023). How I treat cytopenias after CAR T-cell therapy. Blood.

[33] Westin J.R., Oluwole O.O., Kersten M.J., Miklos D.B., Perales M.A., Ghobadi A., Rapoport A.P., Sureda A., Jacobson C.A., Farooq U. (2023). Survival with Axicabtagene Ciloleucel in Large B-Cell Lymphoma. N. Engl. J. Med..

[34] Bishop M.R., Dickinson M., Purtill D., Barba P., Santoro A., Hamad N., Kato K., Sureda A., Greil R., Thieblemont C. (2022). Second-Line Tisagenlecleucel or Standard Care in Aggressive B-Cell Lymphoma. N. Engl. J. Med..

[35] Alarcon Tomas A., Fein J.A., Fried S., Flynn J.R., Devlin S.M., Fingrut W.B., Anagnostou T., Alperovich A., Shah N., Fraint E. (2023). Outcomes of first therapy after CD19-CAR-T treatment failure in large B-cell lymphoma. Leukemia.

[36] Melody M., Gordon L.I. (2024). Sequencing of cellular therapy and bispecific antibodies for the management of diffuse large B-cell lymphoma. Haematologica.

